# Hemispheric dissociation of anxiety and autonomic arousal during lateral visual field viewing: a case report

**DOI:** 10.3389/fnhum.2026.1763515

**Published:** 2026-05-15

**Authors:** Fredric Schiffer

**Affiliations:** 1 Developmental Biopsychiatry Research Program, McLean Hospital, Belmont, MA, United States; 2 Department of Psychiatry, Harvard Medical School, Boston, MA, United States; 3 MindLight, LLC, Newton Highlands, MA, United States; 4 Private Practitioner, Newton Highlands, MA, United States

**Keywords:** autonomic arousal, dual-brain psychology, hemispheric emotional valence, individual differences, lateral visual field stimulation, trauma memory

## Abstract

We report a clinical case illustrating rapid, reversible, and reproducible hemispheric differences in subjective experience and autonomic arousal during lateral visual field viewing. A man in his 40s with longstanding anxiety and depression showed repeatable shifts in affective state, self-appraisal, and appraisal of the clinician when alternately viewing through the right versus left lateral visual field while holding the same shame-evoking interpersonal scenario in mind. Pulse rate measured with a fingertip pulse oximeter was observed to co-occur with these shifts (anxious/shame state ≈105 bpm; calm/secure state ≈85–90 bpm; baseline ≈95 bpm). Across 72 consecutive patients entered into the author’s practice, 89% showed a clinically useful between-field difference in momentary anxiety and 49% showed a marked difference of the type described in this case report. Together with prior findings from split-brain research, Wada studies, fMRI, repetitive transcranial magnetic stimulation (rTMS), and lateral visual field stimulation (LVFS) paradigms, this observation is consistent with the partial functional independence of two hemispheric experiential systems—one of which is more childlike and more affected by past trauma—and with history-dependent meaning as a driver of physiological response. We interpret the case within Dual-Brain Psychology (DBP) and relate the findings to the Emotion-Type Hypothesis (ETH), proposing a distinction between lateralized emotional functions (as characterized by ETH) and hemispheric experiential dominance (as characterized by DBP). On this account, a hemispheric experiential system may function as the dominant organizing self while recruiting emotional and cognitive capacities distributed across both hemispheres. This synthesis may help reconcile population-level associations between emotion type and hemispheric specialization with the within-individual variability in hemispheric emotional valence observed under lateral visual field viewing.

## 1 Introduction

The dominant theories of hemispheric emotional valence (HEV) treat lateralization as a broadly uniform property of the human brain. The valence hypothesis, right-hemisphere hypothesis, and motivational hypothesis each assume a relatively fixed mapping between hemisphere and emotional valence at the species level (Davidson, 1992; Gainotti, 2019, 2024; Harmon-Jones et al., 2010; Killgore and Yurgelun-Todd, 2007; Schiffer et al., 2007). While well-supported by group-average data, these models have difficulty accommodating evidence of substantial individual variability in the direction and degree of hemispheric emotional asymmetry (Coan and Allen, 2004; Hecht, 2014).

An important alternative is the Emotion-Type Hypothesis (ETH) proposed by Ross et al. (1994) and Ross (2021, 2023), which emphasizes qualitative distinctions among emotions rather than simple positive–negative valence mapping. Within this framework, the right hemisphere is preferentially involved in primary, evolutionarily older emotions (e.g., fear, anger, sadness), whereas the left hemisphere plays a greater role in socially mediated emotions that are linguistically structured, reflective, and context-dependent. ETH further predicts differential hemispheric contributions to affective disorders, particularly distinct depressive subtypes (Ross and Rush, 1981; Ross, 2023), and provides one interpretive framework for variable patterns of lateralized emotional response observed in clinical and Wada-test contexts.

Several findings challenge the rigidity of fixed valence models. In a Wada study of 270 epilepsy surgery candidates (562 unilateral amobarbital injections), Stabell et al. (2004) observed clear emotional changes in approximately one-fifth of procedures. Critically, neither euphoria nor dysphoria showed a consistent preference for left- or right-hemisphere anesthesia, a pattern difficult to reconcile with a simple left-positive/right-negative scheme. As we have reviewed in detail elsewhere (Schiffer et al., 2007), the Wada findings indicate that emotional responses depend on multiple factors that are not captured by a fixed hemispheric valence mapping.

Dual-Brain Psychology (DBP) was developed to account for such variability by treating each cerebral hemisphere as supporting a largely complete “self,” with its own emotional, cognitive, and behavioral tendencies (Schiffer, 1997, 2022). Clinically, one hemisphere typically appears more mature, resilient, and optimistic, while the other is more fearful and more burdened by past trauma—but the healthier hemisphere may be either left or right. In a 1995 study using probe auditory evoked potentials, we found that a majority of subjects showed a more negative right hemisphere during recall of upsetting memories, but a substantial minority showed the opposite pattern (Schiffer et al., 1995), anticipating the individual-difference emphasis that has since become central to DBP.

Building on Wittling’s foundational work on lateral visual field stimulation (Wittling and Roschmann, 1993), we demonstrated that LVFS produces reproducible emotional and cognitive differences depending on the visual field viewed, and that the side associated with more troubled emotional experience varies among individuals (Schiffer, 1997; Schiffer et al., 1999). Different HEV lateralities were further associated with differences in grey-matter volume and hippocampal and amygdala measures (Schiffer et al., 2007). Importantly, in Schiffer et al. (1999), the characteristic EEG theta, ear-canal temperature, and anxiety effects were produced by LVF glasses but not by matched-appearance monocular control glasses that simply covered one eye and the very bottom of the other lens, indicating that the lateralizing effect depends on hemifield restriction rather than on monocular occlusion alone. In a subsequent fMRI study at McLean Hospital, LVFS was shown to increase blood flow in the contralateral hemisphere, particularly in occipital, posterior parietal, and dorsolateral prefrontal cortex (Schiffer et al., 2004), directly confirming that the paradigm engages the targeted hemisphere.

More recently, we developed the Computerized Test of Hemispheric Emotional Valence (CTHEV), which uses lateralized presentations of emotional faces and self-reported distress to quantify HEV without direct experimenter involvement. In a sample of 50 right-handed young adults, CTHEV scores varied widely—from clearly left-positive to clearly right-positive—and were systematically related to lateralized volumes of nucleus accumbens, amygdala, and hippocampus, as well as to corpus callosum morphology and white-matter network measures (Schiffer et al., 2022). Across our LVFS, probe evoked potential, and CTHEV studies, approximately 40–70% of individuals demonstrate greater negativity in the left hemisphere—a range that has not yet been characterized systematically but that underscores the degree of individual variability. Together these findings are consistent with the view that HEV is a stable individual-difference dimension, analogous to handedness, rather than a fixed species-typical asymmetry.

**Figure 1:**
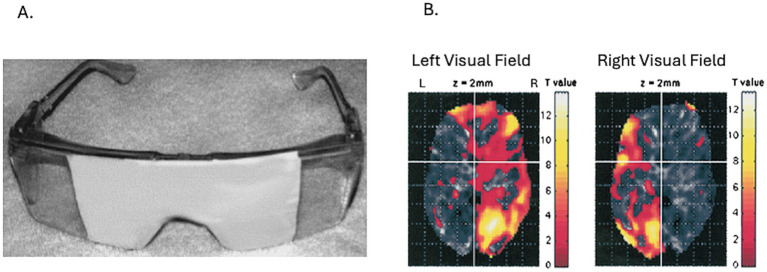
fMRI validation of the LVFS paradigm, reproduced from Schiffer et al. (2004). **(A)** LVFS goggles used in the original fMRI study. **(B)** Group-level SPM99 activation maps for left lateral visual field viewing (left) and right lateral visual field viewing (right), showing robust contralateral activation in occipital, posterior parietal, and dorsolateral prefrontal areas. Reproduced here to document that the lateral visual field paradigm engages the targeted hemisphere directly, providing empirical rather than purely theoretical support for the lateralizing claim.

The present report describes a typical case that illustrates this individual variability particularly clearly, and uses it as the basis for a theoretical synthesis. We propose a distinction between (a) lateralized emotional functions, as characterized by ETH, and (b) hemispheric experiential dominance, as characterized by DBP, arguing that these two constructs operate at different levels of analysis and are mutually compatible. Figure 1 reproduces the published fMRI validation of the LVFS paradigm from Schiffer et al. (2004).

## 2 Case report

### 2.1 Presentation and history

A right-handed man in his 40s presented for psychotherapy with severe anxiety and depression rooted in shame-based traumatic interpersonal early-childhood experiences, longstanding difficulty maintaining employment, and prominent agoraphobic features (reluctance to leave home, avoidance of social situations and dining out). At intake he met criteria for major depressive disorder and generalized anxiety disorder. He had been a long-standing patient seen approximately every two weeks for seven years, with lateral visual field viewing incorporated into psychotherapy sessions throughout treatment to explore within-subject hemispheric differences in subjective state. Medical and neurological histories were notable only for an injury history with some chronic pain. At intake he was being maintained on buprenorphine, escitalopram 20 mg/day, and clonazepam 4 mg/day. The buprenorphine had been initiated by his primary care physician approximately one year before he was first seen by the author, and his dose had been reduced (by the primary care physician) by the time of his first visit; the author judged the buprenorphine therapeutically beneficial for his anxiety and depressive symptoms and continued it during the period covered by this report. The escitalopram had been helpful for an early-morning anger pattern, and was discontinued in mid-2019 with the author’s approval after the patient tolerated several days off without recurrence. The clonazepam was transitioned to lorazepam taken on an as-needed basis, currently 0.5 mg twice daily as needed and substantially reduced from earlier levels. He had a long-standing nicotine habit (initially cigarettes, then vaping) which has more recently been addressed with nicotine-patch tapering.

### 2.2 Intervention

Preferential hemispheric engagement was achieved using the following procedure: the patient held an opaque card over one eye and the medial portion of the other eye’s visual field, restricting visual input to a single lateral visual hemifield and biasing processing toward the contralateral hemisphere. Sessions followed the author’s usual LVFS protocol, in which the patient first views through one lateral visual field while recalling a target memory, reports on subjective state, and then repeats the procedure through the opposite field. Pulse rate was continuously monitored during sessions using a fingertip pulse oximeter (SpO₂ and pulse displayed; only pulse was recorded for this report).

### 2.3 Observed state changes

In a representative session, typical of many repeated sessions with this patient, he was asked to hold a shame-evoking interpersonal scenario in mind while alternately viewing through each lateral visual field. The session timeline is summarized in Table 1.

**Table 1:** Session timeline of lateral visual field viewing (representative session).

Interval	Viewing condition	Prompt held constant	Subjective report/observed state	Pulse (bpm)
Baseline	No occlusion	Recall of embarrassing/abusive interpersonal memory	Baseline state prior to lateral viewing	≈95
1	Right lateral visual field (biases left hemisphere)	Same recalled memory	Anxiety, chest tension, dyspnea; uncertainty about clinician’s opinion of him	≈105
2	Left lateral visual field (biases right hemisphere)	Same recalled memory	Calm, confidence, increased security; positive view of clinician	≈85–90
3	Return to right lateral visual field	Same recalled memory	Distressed state returns rapidly and reproducibly; patient can comment on the contrast	≈105

During right lateral visual field viewing (biasing left-hemisphere engagement), the patient was observed to shift into a somatically anxious state characterized by subjective dyspnea, chills, and a heightened sense of social threat (“worry about being embarrassed, stupid, or a failure”). Pulse rose from a baseline of approximately 95 bpm to approximately 105 bpm after stabilization of the oximeter display. When asked about the therapeutic relationship in this state, he reported uncertainty about the clinician’s opinion of him.

Upon switching to left lateral visual field viewing (biasing right-hemisphere engagement) while holding the same scenario in mind, the patient was observed to shift toward a calmer, more secure state with visible softening of affect (including spontaneous smiling) and increased orientation toward approach (e.g., greater openness to social activity). Pulse decreased to approximately 85–90 bpm. In this state, he reported greater confidence and a clearer perception that the clinician wished him to improve.

These state changes were observed to recur across sessions and occurred within seconds of changing visual field condition. Returning to the right lateral visual field was associated with return of the distressed state. The patient retained continuous awareness of the session content across switches; what appeared to change was the emotional and physiological response to that content, not the content itself. He could observe and comment on the contrast between states, suggesting that the non-dominant hemispheric system remained aware and reflective throughout.

### 2.4 Differential considerations

Differential considerations at the time of assessment included depressive, anxiety, and trauma-related spectrum disorders. The patient’s presentation most closely fit a non-melancholic, anxious depressive syndrome with prominent shame-based cognitions, consistent with the ETH characterization of socially mediated, linguistically structured affective states that preferentially involve left-hemisphere systems (Ross and Rush, 1981; Ross, 2023). Dissociative disorder, psychotic disorder, and organic CNS pathology were considered and judged unlikely based on clinical examination, history, and the ongoing therapeutic relationship.

### 2.5 Follow-up and outcomes

Over the course of treatment, the regulated right-hemisphere-engaged state was used as a scaffold for reappraising shame-based beliefs and developing more compassionate, reality-based interpretations of the patient’s early-childhood experiences. Although the state-dependent contrast under LVFS remains readily elicitable, the patient has shown substantial overall improvement, including reduced intensity and faster recovery from the threat–shame state, increasing capacity to sustain the regulated, secure state outside of sessions, return to consistent functioning, and improved interpersonal engagement. In recent months he has made what he and the author both describe as a quantum leap toward sustained recovery. He has successfully tapered off buprenorphine, has substantially reduced his use of as-needed lorazepam, has remained off escitalopram since 2019, and has substantially reduced or eliminated nicotine use following a period of nicotine-patch tapering. In his own words, the patient writes: “In the past I would be too afraid to get out of bed without a lot of medication…. Through the therapy process I have greatly reduced/stopped medication and found a great deal of relief that has changed the way I live my life…. This process showed me an inner strength I did not know existed.” No adverse effects of lateral visual field viewing were reported over the course of treatment.

### 2.6 Patient perspective

*The patient’s description below uses the two-mind language he learned during treatment; his account is presented in his own words to convey his subjective experience of the process and outcome, not as independent confirmation of the framework. Light copy-editing for typographical errors has been applied; the wording, structure, and substance are the patient’s.*

“Dr. Schiffer’s therapy process is quite exceptional. The initial complaint of physical and emotional distress was validated and recognized in a safe environment. That safe environment allowed for education towards the causes of the distress, such as having two minds. One mind is stuck in a child-like state still trapped in childhood trauma, which results in a counterproductive effort to feel better through self-sabotaging behavior and thinking. Meanwhile, the second mind is more mature, rational, confident, and holds a high self-value. This two-mind experience is learned through a combination of psychotherapy as well as lateralized therapeutic interventions that can change the perception between both minds, resulting in two separate experiences from the same external stimuli.”

“The therapy process then continues to show the benefits of the mature mind’s perception and to have the traumatic mind build trust in the more mature mind. Therefore, the more traumatic mind can calm down, believe in its safety, and trust the mature mind to take control and protect the child-like mind from unnecessary pain. The mature mind can also find empathy towards not only the immature self mind but the immature minds of others and their traumatic experiences.”

“In my patient perspective I truly feel that meeting Dr. Schiffer and working with him will be among the most important things I will ever accomplish. I have never felt so accepting, understanding, rational, and hopeful towards my past, present, and future. I no longer beat myself up with self-sabotaging inner monologue and/or dangerous behavior. The benefits are immeasurable…. The process also went along my own personal timeline. There were no deadlines or race to result. I cannot think of a single harm from this process. This process showed me an inner strength I did not know existed…. I enjoy my patient experience and am grateful to have learned from Dr. Schiffer.”

### 2.7 Prevalence of the LVFS response in the author’s practice

Across 72 consecutive patients entered into the author’s private psychiatric practice, 89% showed a clinically useful between-field difference in emotional state when LVFS was incorporated into the initial assessment, defined as a difference of at least 1 point on a 10-point self-rating of momentary anxiety between right and left lateral visual field viewing while holding a personally relevant evocative scenario in mind. Forty-nine percent showed a marked difference, defined as at least a 3-point between-field difference, of the type described in this case report. This is a retrospective, single-clinician observational series rather than a controlled sample, and the ratings were obtained by the treating clinician during routine care; it is presented here to give the reader a sense of the clinical prevalence of the phenomenon rather than as a formal prevalence estimate.

### 2.8 Additional clinical observations of LVFS-induced state shifts

Table 2 summarizes three additional clinical vignettes from the author’s practice that illustrate the range of behavioral dissociations observable under LVFS, including contrasts in craving, valuation, and transference. All four patients (the case-report patient and these three) showed large experiential differences between the visual fields. These are uncontrolled clinical observations from routine care, not controlled data, and are presented only to show that the phenomenon observed in the main case is not confined to shame-based anxiety but extends across affective, motivational, and relational domains.

**Table 2:** Additional LVFS-induced state shifts from the author’s clinical practice (uncontrolled clinical observations).

Clinical context	LVFS-induced observation	Source
Gambling craving	A patient with a powerful gambling urge, in an imagined auction, offered to pay up to $2,000 to enter an imaginary casino while looking out of the right lateral visual field; through the opposite field he refused to pay anything. This contrast was reproducible within the session across multiple repetitions; after discussion and repeated exposure the between-field difference diminished.	Author’s practice (uncontrolled clinical observation)
Alcohol craving	A patient with alcoholism, in another pretend auction, offered $500 for a pretend beer from the office refrigerator while viewing through the right lateral visual field. Through the opposite field he declined and said, “No, I do not want it,” when offered an actual beer for free.	Author’s practice (uncontrolled clinical observation)
Erotic transference	A patient with a strong erotic transference toward the clinician experienced complete disappearance of the transference when viewing through the right lateral visual field.	Author’s practice (uncontrolled clinical observation)

A recurrent clinical observation across such cases is that when the between-field contrast is elicited repeatedly within a session and each experiential system is engaged in therapeutic discussion, the magnitude of the difference tends to diminish as the more troubled side improves—consistent with therapeutic integration of the two hemispheric stances rather than mere habituation, though the two cannot be disentangled in uncontrolled clinical observation. These clinical observations reinforce that the main case is not isolated, while not themselves constituting controlled evidence.

## 3 Grounding in prior empirical evidence

The present case does not stand in isolation. A sustained program of research has characterized the LVFS paradigm and related methods across physiological, neuroimaging, and clinical-trial designs. This prior empirical base is summarized here because it directly addresses the question of whether the hand-held-card LVFS paradigm used in the case report reliably biases hemispheric engagement.

### 3.1 Validation of the LVFS paradigm

The LVFS paradigm originated with Wittling’s work using a computerized eye-tracking display that presented emotionally evocative films to one hemisphere at a time; different emotional responses were observed to the same film depending on the side of presentation, and the more anxious side could be either left or right (Wittling and Roschmann, 1993). The paradigm does not require tachistoscopic presentation or exact pupil-position control to produce reliable effects; Wittling’s own results were obtained using a continuous-display method.

Following this lead, we demonstrated that similar lateralized affective and physiological effects can be obtained with simpler optical configurations. In Schiffer et al. (1999), 15 participants were evaluated while wearing, in random order, four pairs of glasses: two pairs limiting vision to either the left or right lateral visual field, and two matched-appearance monocular control pairs (taped goggles covering one eye and the very bottom of the other lens, constructed to resemble the experimental glasses in appearance) so that the two pairs differed only in whether hemifield restriction was produced. The LVF glasses produced significant laterality differences in theta EEG (*p* < 0.003), ear-canal temperature (*p* < 0.02), and anxiety ratings; the monocular control glasses did not. This finding directly addresses the concern that LVFS effects might reflect simple monocular occlusion or posture/attention artifacts rather than hemispheric biasing.

In a subsequent fMRI study at McLean Hospital (Schiffer et al., 2004), seven participants wearing LVF goggles viewed alternately to the left and right in a block design. Both conditions produced their strongest activation in the contralateral occipitotemporal and posterior parietal areas, as well as in contralateral dorsolateral prefrontal cortex, directly confirming that the paradigm engages the targeted hemisphere. Figure 1 reproduces the published fMRI result.

### 3.2 LVFS predicts response to lateralized treatment

LVFS-assessed HEV laterality predicted clinical response to left-prefrontal rTMS in two independent samples (Schiffer et al., 2002, 2008). Patients whose left hemisphere was associated with greater negative affect, as assessed by LVFS at baseline, showed poorer response to standard left-sided rTMS—which in those individuals would have been stimulating the already-distressed hemisphere. The predictive relationship replicated across the two studies and supports a personalized, lateralized view of affective neurophysiology with direct implications for treatment selection.

### 3.3 LVFS-guided unilateral transcranial photobiomodulation

LVFS has also been used prospectively to guide lateralized treatment with unilateral transcranial photobiomodulation (UtPBM). In a randomized, double-blind, placebo-controlled trial of 22 patients with opioid cravings, UtPBM applied over the hemisphere with the more positive HEV (as determined by LVFS and a convergent test) produced a 51.0% reduction in opioid craving one week post-treatment versus 15.8% for sham (*p* = 0.004; effect size 0.73; Schiffer et al., 2020). In a second, larger randomized controlled trial at two sites, 39 participants received either active LVFS-guided UtPBM or sham; the active group showed a highly significant treatment × time benefit (*p* < 0.0001) with an effect size of 1.5 at last follow-up on the opioid craving scale (Schiffer et al., 2021). A retrospective clinical series of 42 consecutive opioid-use-disorder patients treated in the author’s practice reported that 62% showed consistently striking clinical benefits, 19% helpful but less remarkable effects, and 19% no noticeable response (Schiffer, 2021). The patient in the present case report also shows a reproducible lateralized response to UtPBM in a direction consistent with his LVFS profile: in his case both LVFS through the left lateral visual field (biasing the right hemisphere by contralateral activation) and UtPBM placed over the right hemisphere preferentially engage the more regulated hemispheric stance.

### 3.4 Convergent neuroanatomical correlates

HEV laterality measured by computerized test correlates with lateralized brain structure and connectivity. In 50 right-handed young adults, CTHEV scores correlated with laterality indices of the nucleus accumbens (*p* = 0.00016), amygdala (*p* = 0.0138), and hippocampus (*p* = 0.031), and with total and segment-specific corpus callosal volumes (Schiffer et al., 2022). These convergent neuroanatomical findings support the interpretation of HEV laterality as a trait-level individual difference with identifiable neural correlates, rather than as a transient or methodological artifact.

### 3.5 Summary

Taken together, the LVFS paradigm has been (a) validated against a matched-appearance monocular control that isolates hemifield restriction as the effective variable (Schiffer et al., 1999); (b) confirmed by fMRI to produce contralateral cortical activation (Schiffer et al., 2004); (c) shown to predict clinical response to a lateralized treatment (rTMS) in two independent samples (Schiffer et al., 2002, 2008); (d) used prospectively to guide a different lateralized treatment (UtPBM) with large effect sizes in two randomized controlled trials (Schiffer et al., 2020, 2021); and (e) linked to lateralized subcortical and callosal neuroanatomy in a separate sample (Schiffer et al., 2022). The paradigm’s reliability is therefore supported by convergent evidence across multiple methods, samples, and outcome domains, and does not depend on precise tachistoscopic timing or exact pupil-position control for its effects.

## 4 Theoretical interpretation

The case motivates a theoretical distinction between two levels of analysis that are frequently conflated in the HEV literature: the lateralization of emotional functions and the lateralization of experiential dominance.

It may be true, as ETH proposes, that one hemisphere preferentially instantiates particular emotional functions, much as the left hemisphere preferentially instantiates language in most right-handers (Ross, 2021, 2023). At the same time, lateral visual field viewing reliably produces persistent and comprehensive personality-level shifts in which one hemispheric experiential system becomes organizationally dominant over the other. In this state the patient speaks fluently, moves both sides of the body, and retains access to Broca’s and Wernicke’s areas regardless of which hemisphere is preferentially engaged; what changes is not cognitive access but experiential stance—the affective coloring, self-evaluation, and interpretive posture brought to the same situation.

We therefore propose that there is a hemispheric experiential system, or hemispheric self, that is capable of recruiting emotional, cognitive, and mnemonic functions distributed across both hemispheres. Whether that self feels immature and anxious or mature and secure in a given moment depends on the developmental history of the hemisphere in which it is preferentially instantiated, not on a fixed species-typical assignment of affect to left or right. A self instantiated in an anxiety-prone hemisphere may recruit an anxiety-related circuit; a self instantiated in a more resilient hemisphere may recruit circuits supporting confidence and security. On this account, ETH correctly identifies population-level tendencies in the hemispheric distribution of emotion types, while DBP characterizes the organizing self that becomes dominant under a given psychological condition. The two accounts operate at different levels of analysis and need not conflict.

This distinction has implications for understanding individual differences in HEV. Within DBP, the variability in left-versus-right negativity observed across our LVFS, evoked-potential, and CTHEV samples is interpreted as reflecting individual differences in which hemisphere has become the seat of the trauma-associated self—a developmental outcome shaped by early experience rather than a fixed anatomical property. HEV is thus analogous to handedness: a population-level tendency (which ETH characterizes) overlying substantial individual variation (which DBP and LVFS research document). Clinical experience working psychotherapeutically with both hemispheric experiential systems consistently shows that the more troubled side is experiencing the present situation through the lens of past trauma (Schiffer, 1998, 2022).

It is important to emphasize that preferential hemispheric engagement under LVFS does not imply anatomical isolation. Patients in this and prior reports retain fluent speech, intact voluntary behavior, and access to lateralized cognitive styles while exhibiting marked shifts in overall affective experience. The patient in the present case demonstrated logical, sequential thinking even when his right hemisphere was preferentially engaged, confirming that cognitive capacities are not confined to a single hemisphere in a simplistic manner.

## 5 Relation to split-brain research

Classic split-brain research has shown that the right hemisphere retains experiential autonomy—including independent affective responses, self-image, and values—even when overt language and behavioral expression come to be dominated by the left hemisphere (Gazzaniga, 1970; Schiffer et al., 1998; Sperry, 1968, 1974). Loss of behavioral expression in the right brains of those patients did not imply loss of experiential organization. The present findings extend this observation to the intact brain, suggesting that hemispheric experiential systems can shift the predominant conscious affective stance without anatomical disconnection, while the non-dominant hemisphere (left or right) remains sufficiently aware to reflect on the contrast between states—as this patient demonstrated.

## 6 Limitations

Several limitations of the present case report should be stated explicitly. First, this is a single clinical case observed within a psychotherapy dyad in which the clinician held prior hypotheses about LVFS outcomes and served as the sole observer, affect rater, and individual recording pulse. Experimenter expectancy, demand characteristics, and therapeutic suggestion cannot be excluded as contributions to the observed state shifts in this single case. We note, however, that the broader empirical studies on which the case rests were conducted outside of the psychotherapy context, generally without the author present during data collection (Schiffer et al., 2002, 2004, 2007, 2008, 2022).

Second, the paradigm did not include within-case control conditions such as central fixation, binocular viewing, sham occlusion, or a neutral (non-trauma-related) cognitive condition; within-case comparisons were limited to alternation between right and left lateral visual fields. The lateralizing effect of the paradigm itself, however, has been tested against an appropriately matched monocular control with objective EEG and physiological outcomes (Schiffer et al., 1999) and against an objective neuroimaging outcome (Schiffer et al., 2004), as detailed in Section 3.

Third, the session in this case report followed a fixed right–left–right sequence; order effects, sequential emotional carry-over, and regression toward baseline cannot be separated from the hemispheric manipulation on the basis of this session alone. In the prior group studies, the order of conditions was randomized or counterbalanced and the lateralizing effects survived (Schiffer et al., 1999, 2002, 2007, 2008).

Fourth, pulse was recorded from a fingertip pulse oximeter as approximate point estimates rather than as continuous ECG or photoplethysmography; heart-rate variability, respiratory rate, and electrodermal activity were not monitored, so interpretation of autonomic findings is limited to the direction and rough magnitude of change. Fifth, the hand-held occluder does not guarantee precise hemifield restriction on a moment-to-moment basis; the paradigm’s lateralizing effect rests on the convergent empirical evidence reviewed in Section 3 rather than on optical precision within this single case. Sixth, the prevalence figures reported in Section 2.7 are from a retrospective clinician-scored series and should be regarded as suggestive rather than as a controlled prevalence estimate.

Finally, the author is the founder and CEO of MindLight, LLC, which develops lateralized-stimulation technologies, holds expired patents on lateralized eyewear, and has a pending patent on a current iteration of lateralized eyewear. This commercial interest is disclosed in full in the Conflict of Interest statement and is noted here because readers should weigh it when interpreting the present observations. The hand-held-card LVFS method described in this case report is not itself the subject of any current or past patent.

Given these limitations, the present case should be read as a hypothesis-generating clinical observation situated within a broader empirical program, rather than as a controlled demonstration of hemispheric-specific causation on its own.

## 7 Clinical implications and future directions

### 7.1 Implications for theories of consciousness

Leading theories of consciousness—including Global Workspace Theory (Baars, 2021; Baars et al., 2013) and Integrated Information Theory (Cea et al., 2023)—are often interpreted as assuming a unified conscious experience at any given moment. The present case and the associated clinical series suggest a more dynamic picture: conscious emotional experience may be characterized by shifting hemispheric predominance, in which one hemispheric experiential system strongly shapes the momentary affective stance while the contralateral system remains active and reflective. The capacity of patients to observe and comment on the contrast between hemispheric states—from within either state—indicates that consciousness is not simply unified by hemispheric predominance but can encompass awareness of its own multiplicity. We offer this as a clinically grounded observation that any general theory of conscious experience will eventually need to accommodate, rather than as an adjudication between specific theoretical frameworks.

### 7.2 Personalizing treatment

In two prior depression studies (Schiffer et al., 2002, 2007), approximately 45–50% of patients showed greater negative affect associated with the left hemisphere rather than the right. This has direct implications for treatment personalization. Mapping an individual’s HEV—using the LVFS assessment or the CTHEV—may help to guide treatment selection for rTMS, targeted psychotherapy, and UtPBM (Schiffer et al., 2002, 2008, 2020, 2021, 2022). The present case further illustrates that the healthier hemispheric state can serve as a clinical resource: in the DBP framework, the more regulated hemispheric stance can function as a scaffold from which to reappraise trauma-based beliefs and build more reality-congruent self-representations.

Because lateral visual field viewing is simple and noninvasive, clinicians may find it useful as an observational tool to explore within-individual hemispheric differences in appropriate settings. Systematic study is needed before broad clinical recommendations can be made; the case report format does not permit prevalence estimates or controlled inference beyond what is already supported by the prior studies cited above.

### 7.3 Testable predictions

The hemispheric-experiential-system account generates several testable predictions:

1. Trait–state mapping: Individuals with a left-negative HEV as assessed independently by CTHEV should show left-hemisphere-engaged distress states under LVFS.
2. Familial clustering: HEV polarity may cluster within families independently of genetics, supporting a developmental and environmental contribution—a prediction testable in family studies.
3. Neural markers: Indices of hemispheric asymmetry—including callosal microstructure, dorsolateral prefrontal dominance, and subcortical volume asymmetries—should converge with HEV polarity as assessed by LVFS or CTHEV.
4. Treatment response: Patients whose HEV is mapped prior to rTMS or UtPBM and who receive lateralized stimulation consonant with their HEV profile should show better clinical outcomes than those receiving standard (non-individualized) lateralized stimulation.

## 8 Conclusion

This case illustrates a reproducible form of hemispheric dissociation: when processing is biased toward one hemisphere via lateral visual field viewing, the patient was observed to shift rapidly between distinct affective and self-referential states, accompanied by directionally consistent autonomic changes. Taken together with prior findings from LVFS paradigms, split-brain research, and lateralized neuromodulation, these observations are consistent with the view that each hemisphere can function as a partially independent experiential system under specific conditions.

We have proposed a distinction between lateralized emotional functions, as characterized by ETH (Ross, 2021, 2023), and hemispheric experiential dominance, as characterized by DBP (Schiffer, 2022). These constructs operate at different levels of analysis—the first characterizing population-level tendencies in the hemispheric distribution of emotion types, the second characterizing the organizing self that becomes dominant under a given psychological condition—and we argue that they are mutually compatible and jointly useful for understanding hemispheric emotional asymmetry. Clinically, these findings motivate patient-specific interventions that respect hemispheric differences in meaning, affect, and agency, and they offer a concrete bedside therapeutic method for beginning to explore such differences in psychotherapy and experimental settings.

## Data Availability

The original contributions presented in the study are included in the article/supplementary material, further inquiries can be directed to the corresponding author/s.

## References

[ref1] Baars B. J. (2021). On Consciousness: Science and Subjectivity—Updated Works on Global Workspace Theory. New York, NY: Nautilus Press.

[ref2] Baars B. J. Franklin S. Ramsoy T. Z. (2013). Global workspace dynamics: cortical “binding and propagation” enables conscious contents. Front. Psychol. 4:200. doi: 10.3389/fpsyg.2013.00200, 23974723 PMC3664777

[ref3] Cea G. Sanz L. R. D. Rosanova M. (2023). Integrated information theory. Curr. Biol. 33, R989–R994.

[ref4] Coan J. A. Allen J. J. B. (2004). Frontal EEG asymmetry as a moderator and mediator of emotion. Biol. Psychol. 67, 7–49. doi: 10.1016/j.biopsycho.2004.03.002, 15130524

[ref5] Davidson R. J. (1992). Anterior cerebral asymmetry and the nature of emotion. Brain Cogn. 20, 125–151. doi: 10.1016/0278-2626(92)90065-T, 1389117

[ref6] Gainotti G. (2019). Emotions and the right hemisphere: can new data clarify old models? Neuroscientist 25, 258–270. doi: 10.1177/1073858418785342, 29985120

[ref7] Gainotti G. (2024). Emotions related to threatening events are mainly linked to the right hemisphere. J. Psychiatry Neurosci. 49, E208–E211. doi: 10.1503/jpn.240002, 38816030 PMC11149614

[ref8] Gazzaniga M. S. (1970). The Bisected brain. New York, NY: Appleton-Century-Crofts.

[ref9] Harmon-Jones E. Gable P. A. Peterson C. K. (2010). The role of asymmetric frontal cortical activity in emotion-related phenomena: a review and update. Biol. Psychol. 84, 451–462. doi: 10.1016/j.biopsycho.2009.08.010, 19733618

[ref10] Hecht D. (2014). Cerebral lateralization of pro- and anti-social tendencies. Experimental Neurobiology 23, 1–27. doi: 10.5607/en.2014.23.1.1, 24737936 PMC3984952

[ref11] Killgore W. D. S. Yurgelun-Todd D. A. (2007). The right-hemisphere and valence hypotheses: could they both be right (and sometimes left)? Soc. Cogn. Affect. Neurosci. 2, 240–250. doi: 10.1093/scan/nsm020, 18985144 PMC2569811

[ref12] Ross E. D. (2021). Differential hemispheric lateralization of emotions and related display behaviors: emotion-type hypothesis. Brain Sci. 11:1128. doi: 10.3390/brainsci11081034, 34439653 PMC8393469

[ref13] Ross E. D. (2023). Affective prosody and its impact on the neurology of language, depression, memory and emotions. Brain Sci. 13:1496. doi: 10.3390/brainsci13111572 38002532 PMC10669595

[ref14] Ross E. D. Homan R. Buck R. W. (1994). Differential hemispheric lateralization of primary and social emotions: implications for developing a comprehensive neurology for emotions, repression, and the subconscious. Neuropsychiatr. Neuropsychol. Behav. Neurol. 7, 1–19.

[ref15] Ross E. D. Rush A. J. (1981). Diagnosis and neuroanatomical correlates of depression in brain-damaged patients: implications for a neurology of depression. Arch. Gen. Psychiatry 38, 1344–1354. doi: 10.1001/archpsyc.1981.01780370046005, 7316678

[ref16] Schiffer F. (1997). Affect changes observed with right versus left lateral visual field stimulation in psychotherapy patients: possible physiological, psychological, and therapeutic implications. Compr. Psychiatry 38, 289–295. doi: 10.1016/S0010-440X(97)90062-6, 9298322

[ref17] Schiffer F. (1998). Of two minds: The Revolutionary Science of dual-brain Psychology. New York, NY: The Free Press.

[ref18] Schiffer F. (2021). Unilateral transcranial photobiomodulation for opioid addiction in a clinical practice: a clinical overview and case series. J. Psychiatr. Res. 133, 134–141. doi: 10.1016/j.jpsychires.2020.12.004, 33340792

[ref19] Schiffer F. (2022). Dual-brain psychology: a novel theory and treatment based on cerebral laterality and psychopathology. Front. Psychol. 13:986374. doi: 10.3389/fpsyg.2022.986374, 36337511 PMC9627780

[ref20] Schiffer F. Anderson C. M. Teicher M. H. (1999). Electroencephalogram, bilateral ear temperature, and affect changes induced by lateral visual field stimulation. Compr. Psychiatry 40, 221–225. doi: 10.1016/S0010-440X(99)90007-X, 10360618

[ref21] Schiffer F. Glass I. Lord J. Teicher M. H. (2008). Prediction of clinical outcomes from rTMS in depressed patients with lateral visual field stimulation: a replication. J. Neuropsychiatry Clin. Neurosci. 20, 194–200. doi: 10.1176/jnp.2008.20.2.194, 18451190

[ref22] Schiffer F. Khan A. Bolger E. Flynn E. Seltzer W. P. Teicher M. H. (2021). An effective and safe novel treatment of opioid use disorder: unilateral transcranial photobiomodulation. Front. Psych. 12:713686. doi: 10.3389/fpsyt.2021.713686, 34447323 PMC8382852

[ref23] Schiffer F. Khan A. Ohashi K. Hernandez Garcia L. C. Anderson C. M. Nickerson L. D. . (2022). Individual differences in hemispheric emotional valence by computerized test correlate with lateralized differences in nucleus accumbens, hippocampal, and amygdala volumes. Psychol. Res. Behav. Manag. 15, 1371–1384. doi: 10.2147/PRBM.S357138, 35673325 PMC9167593

[ref24] Schiffer F. Mottaghy F. M. Pandey Vimal R. L. Renshaw P. F. Cowan R. Pascual-Leone A. . (2004). Lateral visual field stimulation reveals extrastriate cortical activation in the contralateral hemisphere: an fMRI study. Psychiatry Res. 131, 1–9. doi: 10.1016/j.pscychresns.2004.01.002, 15246450

[ref25] Schiffer F. Reichmann W. Flynn E. Hamblin M. R. McCormack H. (2020). A novel treatment of opioid cravings with an effect size of .73 for unilateral transcranial photobiomodulation over sham. Front. Psych. 11:827. doi: 10.3389/fpsyt.2020.00827, 32973577 PMC7466767

[ref26] Schiffer F. Stinchfield Z. Pascual-Leone A. (2002). Prediction of clinical response to transcranial magnetic stimulation for depression by baseline lateral visual-field stimulation. Neuropsychiatry Neuropsychol. Behav. Neurol. 15, 18–27., 11877548

[ref27] Schiffer F. Teicher M. Anderson C. Tomoda A. Polcari A. Navalta C. . (2007). Determination of hemispheric emotional valence in individual subjects: a new approach with research and therapeutic implications. Behav. Brain Funct. 3:13. doi: 10.1186/1744-9081-3-13, 17341309 PMC1820787

[ref28] Schiffer F. Teicher M. H. Papanicolaou A. C. (1995). Evoked potential evidence for right brain activity during the recall of traumatic memories. J. Neuropsychiatry Clin. Neurosci. 7, 169–175., 7626959 10.1176/jnp.7.2.169

[ref29] Schiffer F. Zaidel E. Bogen J. Chasan-Taber S. (1998). Different psychological status in the two hemispheres of two split-brain patients. Neuropsychiatry Neuropsychol. Behav. Neurol. 11, 151–156. 9742514

[ref30] Sperry R. W. (1968). Hemisphere deconnection and unity in conscious awareness. Am. Psychol. 23, 723–733. doi: 10.1037/h0026839, 5682831

[ref31] Sperry R. W. (1974). “Lateral specialization in the surgically separated hemispheres,” in The Neurosciences: Third Study program, eds. Schmitt F. O. Worden F. G. (Cambridge, Massachusetts: MIT Press), 5–19.

[ref32] Stabell K. E. Andresen S. Bakke S. J. Bjornaes H. Borchgrevink H. M. Heminghyt E. . (2004). Emotional responses during unilateral amobarbital anesthesia: differential hemispheric contributions? Acta Neurol. Scand. 110, 313–321. doi: 10.1111/j.1600-0404.2004.00329.x, 15476460

[ref33] Wittling W. Roschmann R. (1993). Emotion-related hemisphere asymmetry: subjective emotional responses to laterally presented films. Cortex 29, 431–448. doi: 10.1016/S0010-9452(13)80252-3, 8258284

